# *Citrus aurantium *flavonoids inhibit adipogenesis through the Akt signaling pathway in 3T3-L1 cells

**DOI:** 10.1186/1472-6882-12-31

**Published:** 2012-04-03

**Authors:** Gon-Sup Kim, Hyoung Joon Park, Jong-Hwa Woo, Mi-Kyeong Kim, Phil-Ok Koh, Wongi Min, Yeoung-Gyu Ko, Chung-Hei Kim, Chung-Kil Won, Jae-Hyeon Cho

**Affiliations:** 1 Institute of Life Science, College of Veterinary Medicine, Gyeongsang National University, Jinju, South Korea; 2 Animal Genetic Resources Station, National Institute of Animal Science, RAD, Namwon, South Korea; 3 Department of Animal Science & Biotechnology, Gyeongnam National University of Science and Technology, Jinju, South Korea; 4 Department of Anatomy and Developmental Biology, College of Veterinary Medicine, Gyongsang National University, 900 Gajwa-dong, Jinju 660-701, South Korea

## Abstract

**Background:**

Obesity is a health hazard that is associated with a number of diseases and metabolic abnormalities, such as type-2 diabetes, hypertension, dyslipidemia, and coronary heart disease. In the current study, we investigated the effects of *Citrus aurantium *flavonoids (CAF) on the inhibition of adipogenesis and adipocyte differentiation in 3T3-L1 cells.

**Methods:**

During adipocyte differentiation, 3T3-L1 cells were treated with 0, 10, and 50 μg/ml CAF, and then the mRNA and protein expression of adipogenesis-related genes was assayed. We examined the effect of CAF on level of phosphorylated Akt in 3T3-L1 cells treated with CAF at various concentrations during adipocyte differentiation.

**Results:**

The insulin-induced expression of C/EBPβ and PPARγ mRNA and protein were significantly down-regulated in a dose-dependent manner following CAF treatment. CAF also dramatically decreased the expression of C/EBPα, which is essential for the acquisition of insulin sensitivity by adipocytes. Moreover, the expression of the aP2 and FAS genes, which are involved in lipid metabolism, decreased dramatically upon treatment with CAF. Interestingly, CAF diminished the insulin-stimulated serine phosphorylation of Akt (Ser473) and GSK3β (Ser9), which may reduce glucose uptake in response to insulin and lipid accumulation. Furthermore, CAF not only inhibited triglyceride accumulation during adipogenesis but also contributed to the lipolysis of adipocytes.

**Conclusions:**

In the present study, we demonstrate that CAF suppressed adipogenesis in 3T3-L1 adipocytes. Our results indicated that CAF down-regulates the expression of C/EBPβ and subsequently inhibits the activation of PPARγ and C/EBPα. The anti-adipogenic activity of CAF was mediated by the inhibition of Akt activation and GSK3β phosphorylation, which induced the down-regulation of lipid accumulation and lipid metabolizing genes, ultimately inhibiting adipocyte differentiation.

## Background

Obesity is a worldwide epidemic, and there are multiple obesity-associated health problems, which include type-2 diabetes, hypertension, and cardiovascular disease [1]. Obesity is caused by an increase in adipose tissue mass, which results from the multiplication of fat cells through adipogenesis and the increased deposition of cytoplasmic triglycerides [2]. Adipocytes are highly specialized cells that play a critical role in regulating lipid metabolism and energy balance, and they are associated with adipose tissue mass [3]. However, excess adipose tissue leads to obesity and its associated diseases, which causes serious health problems.

Adipogenesis is the process by which an undifferentiated preadipocyte is converted to a fully differentiated adipocyte [3]. Lipid accumulation increases throughout the adipogenic process, and it is regulated by genetic and growth factors [4]. The programmed differentiation of preadipocytes is accompanied by an increase in the expression of various transcription factors and adipocyte-specific genes. 3T3-L1 cells develop from spindle-shaped fibroblast cells into larger, spherical cells, accumulate large triglyceride droplets, and activate adipogenic marker genes. The 3T3-L1 cell line is one of the most reliable models for the study of the conversion of preadipocytes into adipocytes [5]. During differentiation, the action of adipogenic genes, which include C/EBPs and PPARγ, induces adipogenesis [6,7]. Members of the CCAAT-enhancer binding protein family (C/EBP-α, -β and -δ) play important roles in adipogenesis [7]. C/EBPβ is expressed early in the adipocyte differentiation program, and it initiates mitotic clonal expansion (MCE) [8]. In response to an adipogenic induction, C/EBPβ and -δ are first activated to promote PPARγ and C/EBPα expression [7]. The transcription factor PPARγ is a master regulator of adipocyte differentiation, and its activation is both necessary and sufficient for adipocyte differentiation [6]. The activation of C/EBPα and PPARγ leads to terminal differentiation through their subsequent transactivation of adipocyte-specific genes such as fatty acid binding protein 4 (aP2), lipoprotein lipase (LPL), and fatty acid synthase (FAS) [6,9].

The serine/threonine kinase Akt plays an essential role in adipocyte differentiation. Mouse embryonic fibroblasts (MEFs) lacking Akt failed to differentiate into mature adipocytes [10], and an RNAi-mediated decreased in Akt was found to block the differentiation of 3T3-L1 cells [11]. Moreover, the overexpression of constitutively active Akt in 3T3-L1 adipocytes was found to promote glucose uptake and adipocyte differentiation [12]. Akt phosphorylates and regulates a large number of substrates that are involved in a diverse array of biological processes [13], many of which could contribute to the role of Akt in driving adipocyte differentiation. GSK3β is a critical downstream signaling protein in the phosphoinositide 3-kinase (PI3K)/Akt pathway. Insulin signaling activates Akt through PI3K and induces the serine/threonine phosphorylation of downstream targets, such as GSK3β [14]. GSK3β is a critically important protein kinase in adipocyte differentiation because it phosphorylates a number of substrates, including the transcription factor beta-catenin, CCAAT-enhancer binding protein beta (C/EBPβ), and C/EBPα, and promotes glycogen synthesis (GS), which regulates their function [15,16].

*Citrus aurantium *L. (CAL) produces many compounds, such as flavonoids, limonoids, and polyphenols [17,18]. The major flavonoids isolated from CAL include hesperidin, naringenin, and nobiletin, and naringenin and hesperetin have been used to treat cardiovascular diseases [18,19]. Additionally, other studies have shown that CAL extracts exhibit lipolytic activity in human adipocytes [20], and *Citrus aurantium*-induced lipolytic activity has been reported to reduce body fat mass in obese humans [21]. However, the effects of *Citrus aurantium *on adipogenesis are not fully understood.

In the present study, the effect of CAF on adipocyte differentiation in 3T3-L1 cells was investigated by measuring lipid accumulation and evaluating the expression levels of adipocyte marker genes and their target genes. Moreover, we examined whether Akt and GSK3β activation is critical for the anti-adipogenic functions of CAF to better understand the specific mechanisms mediating CAF function. Our results show that the CAF treatment of 3T3-L1 adipocytes inhibits the insulin-induced phosphorylation of Akt at Ser473 and of GSK3β at Ser9 and reduces insulin-stimulated PPARγ, C/EBPβ, and C/EBPα expression, thus suppressing adipocyte differentiation.

## Methods

### Cell culture

Cell culture media and supplements were obtained from Sigma-Aldrich (St. Louis, MO, USA). Mouse 3T3-L1 preadipocytes were grown in Dulbecco's modified eagle medium (DMEM) high glucose containing 10% calf serum at 37°C in a humidified atmosphere of 5% CO_2_. The cells were subcultured after reaching 80% confluence. To induce adipogenesis, 3T3-L1 cells were cultured until confluent, and 1 day after reaching confluence (day 0), the culture medium was exchanged with a differentiation/induction medium (DMII) containing 100 mM insulin, 0.5 mM 3-isobutyl-1-methylxanthine, 100 μM indomethasone, 0.25 μM dexamethasone and 10% fetal bovine serum in DMEM. The DMII was changed every 2 days. The 3-isobutyl-1-methylxanthine, dexamethasone, indomethasone, and Oil-red O were purchased from Sigma-Aldrich (St. Louis, MO, USA). CAF was added to the culture medium containing adipocytes at day 0. Cells were treated with 0, 10, or 50 μg/ml of CAF. After treatment with CAF for 4 or 6 days, the 3T3-L1 adipocytes were lysed for experimental analysis. CAF cytotoxicity was evaluated by 3-(4,5-demethylthiazol-2-yl)-2,5-diphenyltetrazolium bromide (MTT) assay for cell viability.

### Preparation of *Citrus aurantium *Flavonoids

The CAF extracts were supplied by Dr. Shin (Department of Chemistry, Gyeongsang National University) and Kim (Department of Biochemistry, Gyeongsang National University). CAL consists of many flavonoids, naringin, hesperidin, poncirin, isosiennsetin, hexamethoxyflavone, sineesytin, hexamethoxyflavone, tetramrthnl-o-isoscutellaeein, nobiletin, heptamethoxyflavone, 3-hydoxynobiletin, tangeretin, hydroxypentamethoxyflavone, and hexamethoxyflavone [22,23].

### Oil-red O staining

Cells were treated either with CAF (10 μg/ml or 50 μg/ml) or vehicle in differentiation medium from days 0-6 of adipogenesis. On day 4 or 6, cells were stained with Oil-red O. For Oil-red O staining, cells were washed gently with phosphate-buffered saline (PBS) and stained with filtered Oil-red O solution (in 60% isopropanol and 40% water) for 30 min. After staining the lipid droplets, the Oil-red O staining solution was removed, and the plates were rinsed with water and dried. The stained lipid droplets were viewed on an Olympus microscope (Tokyo, Japan).

### Measurement of triglyceride content

Cellular triglyceride contents were measured using a commercially available triglyceride assay kit (Sigma-Aldrich, St. Louis, MO, USA) according to the manufacturer's instructions. Adipocytes differentiated for 4 or 6 days were treated with CAF at concentrations of 0, 10, and 50 μg/ml in 6-well plates. To analyze the cellular triglyceride content, cells were washed with PBS and then scraped into 200 μl PBS and homogenized by sonication for 1 min. The total triglycerides in the lysates were measured using the assay kits.

### RT-PCR

Total RNA was isolated from 3T3-L1 adipocytes using Trizol reagent (Invitrogen, CA, USA). A 1 μg sample of total RNA was subjected to first-strand cDNA synthesis with oligo (deoxythymidine) primers and Superscript II reverse transcriptase (Invitrogen, CA, USA). The target cDNA was amplified using the following sense and antisense primers: sense 5'-GACTACGCAACACACGTGTAACT-3' and antisense 5'-CAAAACCAAAAACATCAACAACCC-3' for C/EBPβ; sense 5'-TTT-TCA-AGG-GTG-CCA-GTT-TC-3' and antisense 5'-AAT-CCT-TGG-CCC-TCT-GAG-AT-3' for PPARγ; sense 5'-TCC-AAG-GAA-GCC-TTT-GAG-AA-3' and antisense 5'-CCA-TCC-TCA-GTC-CCA-GAA-AA-3' for LPL; β-actin was detected as a control using sense (5'- GACAACGGCTCCGGCATGTGCAAAG-3') and antisense (5'-TTCACGGTTGGCCTTAGGGTTCAG-3') primers under the same conditions.

### Western blot analysis

Western blotting was performed according to standard procedures [11]. Briefly, cells were lysed in a buffer containing 50 mM Tris-HCl (pH 8.0), 0.4% Nonidet P-40, 120 mM NaCl, 1.5 mM MgCl_2_, 0.1% SDS, 2 mM phenylmethylsulfonyl fluoride, 80 μg/ml leupeptin, 3 mM NaF and 1 mM DTT. Cell lysates were separated by 10% SDS-polyacrylamide gel electrophoresis, transferred onto a polyvinylidene fluoride membrane (Amersham Pharmacia, England, UK), blocked with 5% skim milk and hybridized with primary antibodies. PPARγ, C/EBPβ, C/EBPα, aP2, Akt, FAS and GSK3β antibodies were from Cell Signaling and the monoclonal β-actin antibody was from Chemicon. HRP-labeled mouse anti-rabbit IgG was obtained from Jackson ImmunoResearch. After incubation with horseradish-peroxidase-conjugated secondary antibody at room temperature, immunoreactive proteins were detected using a chemiluminescent ECL assay kit (Amersham Pharmacia, UK) according to the manufacturer's instructions.

### Lipolysis assay

To obtain fully differentiated 3T3-L1 adipocytes, confluent cells were induced to differentiate in DMII medium for 6 days. The differentiated 3T3-L1 adipocytes were then incubated with 0, 10, or 50 μg/ml of CAFs for 24 hours. The conditioned media was then removed from each well and assayed for glycerol content with free glycerol determination kit (Sigma-Aldrich, St. Louis, MO, USA).

### Statistical analysis

Data are expressed as the means ± SD. Comparison between groups made by ANOVA variance analysis, and significance was analyzed by Duncan's multiple range tests. Differences of *p *< 0.05 were considered to be statistically significant.

## Results

### *Citrus aurantium *flavonoids (CAF) inhibits 3T3-L1 adipocyte differentiation

**Figure 1:**
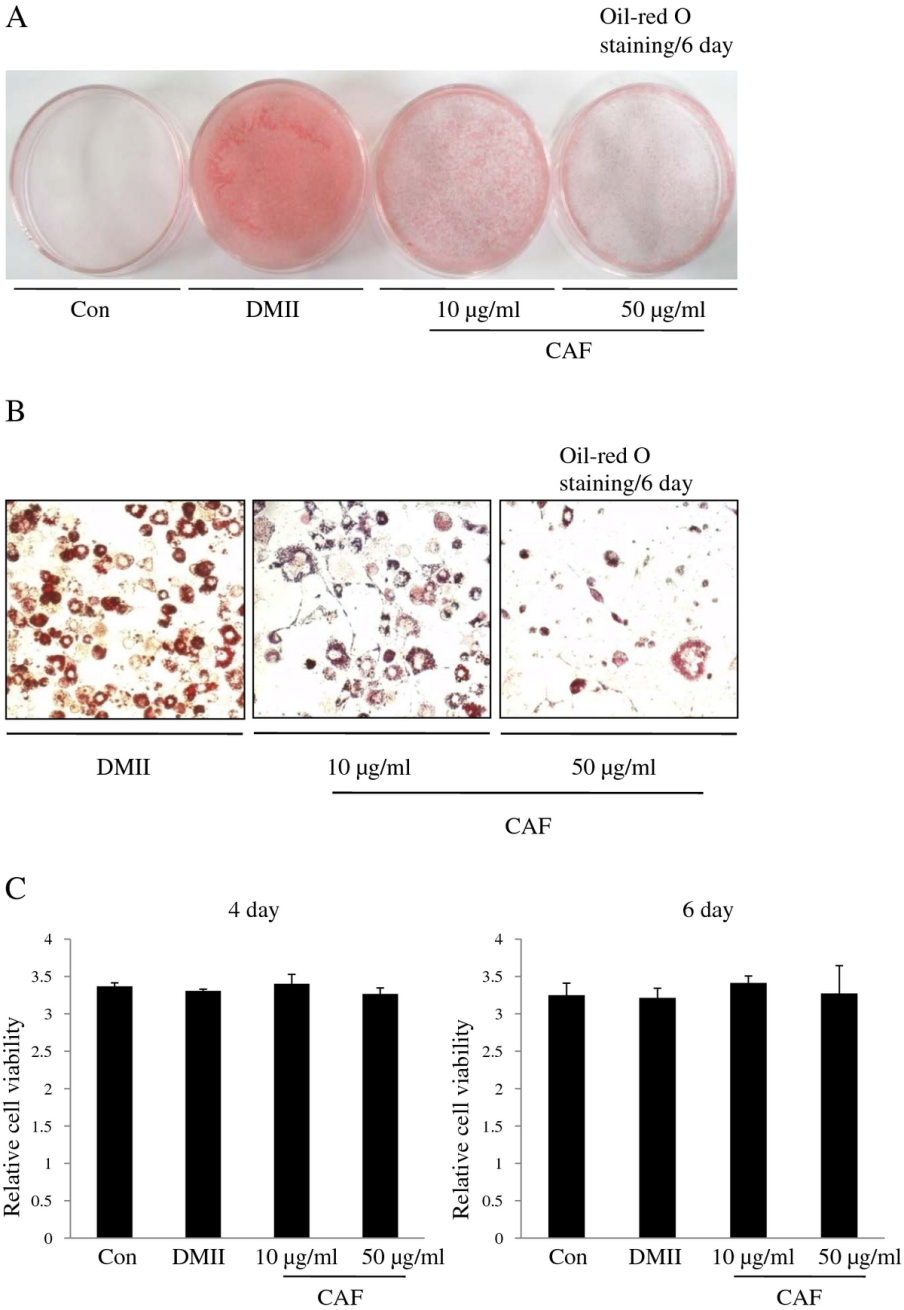
**Effect of CAF on lipid accumulation of 3T3-L1 adipocytes**. Confluent 3T3-L1 preadipocytes differentiated into adipocytes in medium containing different concentrations of CAF for 6 days (from day 0 to 6). Con, 3T3-L1 preadipocytes; DMII, fully differentiated adipocytes; 10 μg/ml, fully differentiated adipocytes (DMII + 10 μg/ml CAF); 50 μg/ml, fully differentiated adipocytes (DMII + 50 μg/ml CAF). (A, B) Inhibitory effects of CAF on lipid accumulation in 3T3-L1 adipocytes. The intracellular lipid accumulation quantified by Oil-red O staining and also optically observed by an inverted microscope. Oil-red O staining assay for lipid droplets was performed on day 6 after induction of differentiation. (C) Effect of CAF on cell viability in preadipocyte and differentiated adipocytes. These data were presented as relative cell viability values. Data are mean ± SD values of at least three independent experiments. Each experiment was performed in triplicate.

To examine the effect of CAF on adipocyte differentiation, 3T3-L1 preadipocytes were treated with various concentrations (0, 10, or 50 μg/ml) of CAF in the presence of DMII (0.5 mM 3-isobutyl-1-methylxanthine, 100 μM indomethacin, 0.25 μM dexamethasone and 100 mM insulin) or DMII alone. The differentiation medium was exchanged with medium containing the DMII mixture every two days. The medium that contained CAF accumulated lipid droplets from day 0 to day 6, and the droplets were visualized by Oil-red O staining on day 6. On day 6 post-induction, oil droplets were not visible in the medium of undifferentiated 3T3-L1 cells, but many lipid droplets were visible in that of the fully differentiated cells treated with DMII. The presence of lipid droplets was used as a marker of lipid accumulation. Figure 1 shows that CAF displays anti-adipogenic properties, which is indicated by the decreased levels of Oil-red O staining at differentiation day 6 (Figure 1A). Microscopic observations of the Oil-red O staining revealed a reduction in the amount of lipid droplets with increasing concentrations of CAF in a dose-dependent manner (Figure 1B). The highest CAF concentration used (50 μg/ml) strongly inhibited the differentiation of 3T3-L1 preadipocytes into adipocytes and prevented lipid accumulation. The inhibitory effects of CAF on triglyceride accumulation during adipogenesis were also dose-dependent. The MTT assay was used to determine the effect of CAF on the proliferation of 3T3-L1 preadipocytes. Treatment with CAF had no significant inhibitory effects on cell viability in cells treated with 10 μg/ml or 50 μg/ml CAF on day 6 post-induction (Figure 1C). These results demonstrate that CAF strongly blocks adipocyte differentiation in 3T3-L1 cells without affecting viability or proliferation.

### The effect of CAF on the expression of adipocyte-specific genes during adipocyte differentiation

**Figure 2:**
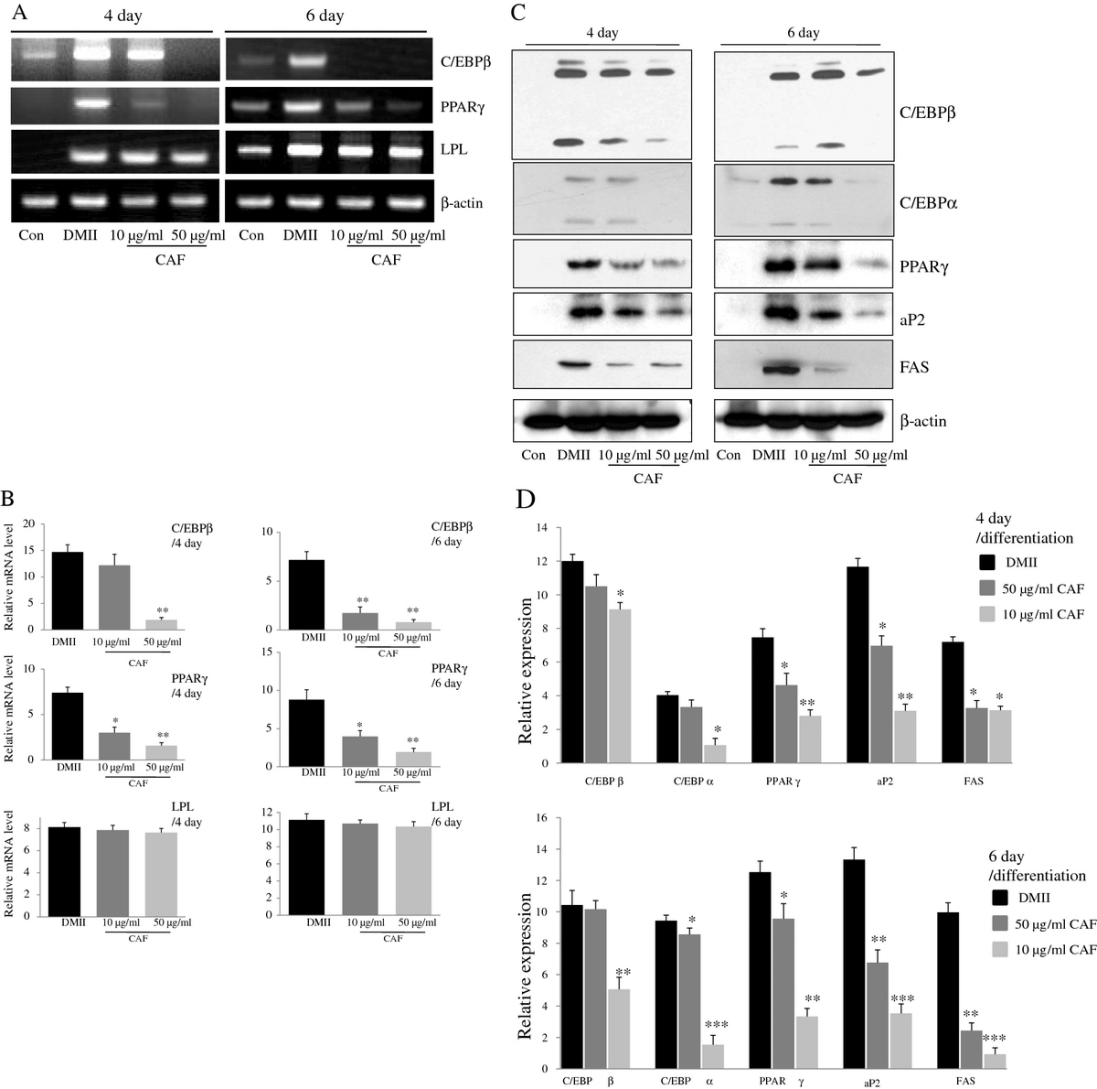
**Effect of CAF on the expression of adipogenesis-related genes in 3T3-L1 adipocytes**. 3T3-L1 cells were differentiated in the absence or presence of CAF for 4 or 6 days. (A) CAF inhibited the expression of adipocyte-specific transcription factors, C/EBPβ and PPARγ mRNA during adipocyte differentiation. The differentiation of 3T3-L1 preadipocytes was induced by DMII medium in the absence or presence of 10 and 50 μg/ml CAF. Total RNA was isolated from 3T3-L1 adipocytes at day 4 and day 6 after induction of differentiation. The expression of C/EBPβ and PPARγ was examined by RT-PCR. (B) The relative expression of adipocyte-specific genes after treating CAF for 4 or 6 days. All gene expressions were normalized using β-actin as a reference gene. The data shown are representative of three independent experiments. (*) *p *< 0.05, (**) *P *< 0.01 versus the control (DMII) group at each gene expression. (C) CAF inhibited the expression of adipogenesis-related genes in 3T3-L1 adipocytes. Total cell lysates were isolated from 3T3-L1 adipocytes at day 4 and day 6 after induction of differentiation. Immunoblotting analysis was performed as described in Materials and Methods. (D) The relative expression of adipogenesis-related genes after treating CAF for 4 or 6 days. The data shown are representative of three independent experiments. (*) *p *< 0.05, (**) *P *< 0.01, (***) *P *< 0.001 versus the control (DMII) group at each gene expression.

To investigate the effects of CAF on the differentiation of 3T3-L1 preadipocytes, 3T3-L1 cells were differentiated in DMII medium containing 10 μg/ml or 50 μg/ml CAF for 6 days. Adipogenesis is accompanied by the increased expression of various transcription factors and adipocyte-specific genes. PPARγ and C/EBPβ are two key transcription factors that are involved in adipogenesis. RT-PCR analysis revealed that the expression of C/EBPβ and PPARγ mRNA was highly induced at day 4 or 6 of adipogenesis, whereas the expression of those genes was significantly decreased in cells treated with CAF (Figure 2A and 2B). However, LPL mRNA expression was not changed significantly by CAF treatment (Figure 2A and 2B). Western blot analysis was conducted to further examine the effect of CAF on the regulation of adipogenic markers at the protein level. As expected, the expression of adipogenic markers, which included PPARγ, C/EBPα, and C/EBPβ, was significantly up-regulated during differentiation (Figure 2C and 2D). However, the expression of PPARγ, C/EBPα, and C/EBPβ was significantly decreased in a time- and dose-dependent manner by the continuous CAF treatment in 3T3-L1 cells, although the expression of C/EBPα and C/EBPβ was only slightly decreased in the cells treated with 10 μg/ml CAF (Figure 2C and 2D). We further investigated whether the CAF-induced reduction of PPARγ and C/EBPα regulated the expression of their target genes, such as FAS and aP2. Treatment of 3T3-L1 adipocytes with CAF significantly down-regulated the expression of aP2 and FAS genes in a dose-dependent manner compared to treatment with DMII without CAF (Figure 2C and 2D).

### The effect of CAF on the regulation of Akt and GSK3β during adipocyte differentiation

**Figure 3:**
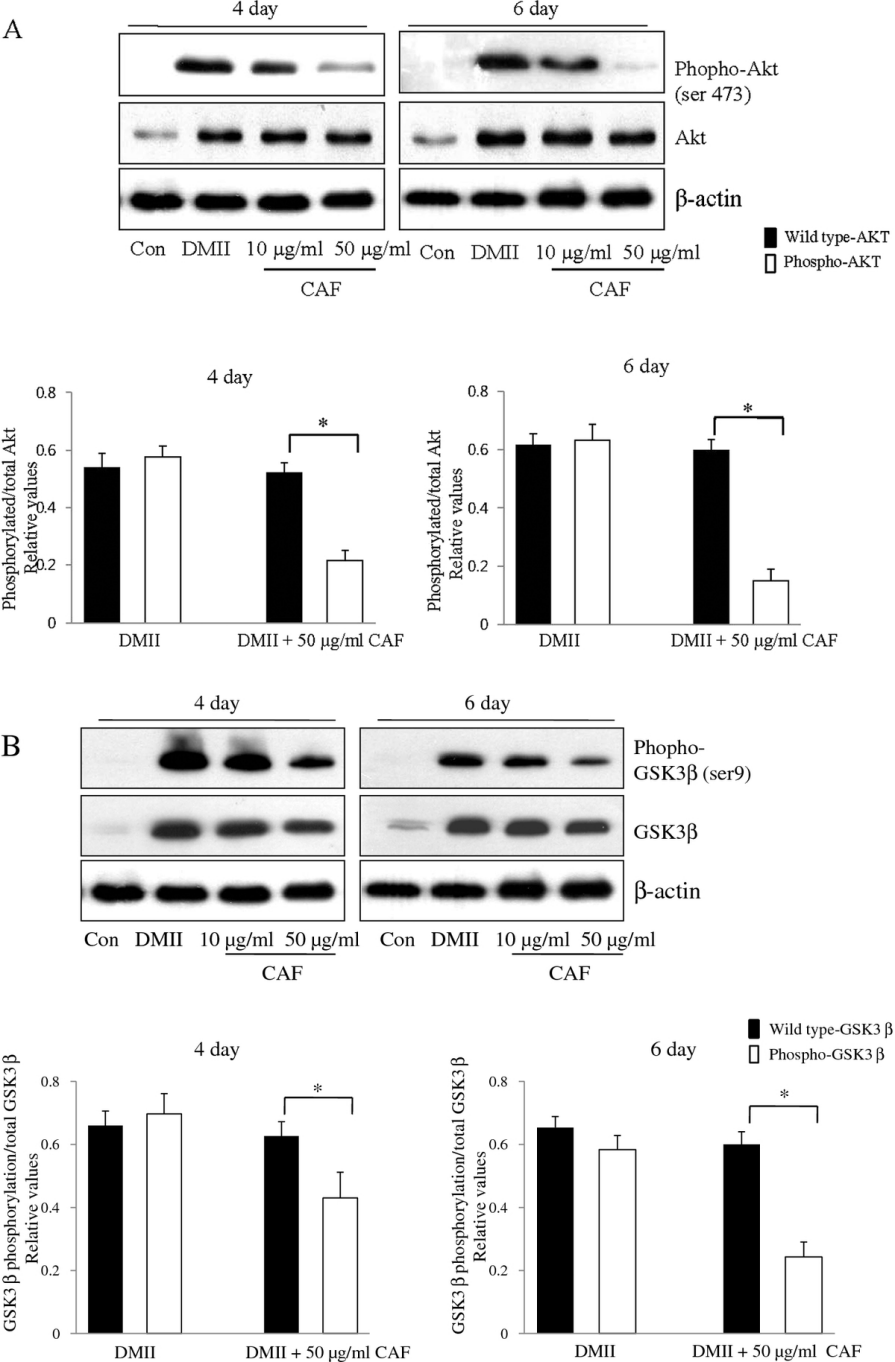
**Effect of CAF on phosophorylation of Akt and GSK3β during 3T3-L1 differentiation**. 3T3-L1 preadipocytes were differentiated in the absence or in the presence of CAF for 6 days. (A) Immunoblotting analysis for wild type Akt and pAkt (Ser 473) was described under Materials and Methods. These experiments were conducted as independent experiments in triplicate. Data represent the mean ± SD. (*) *p *< 0.05. (B) Immunoblotting analysis for wild type GSK3β and pGSK3β (Ser-9) was described under Materials and Methods. These experiments were performed as independent experiments in triplicate. Data represent the mean ± SD. (*) *p *< 0.05.

To determine whether CAF changed the phosphorylation levels of molecules that were downstream of insulin signaling, 3T3-L1 adipocytes were treated with DMII alone or with DMII and CAF. Lysates were collected and immunoblotted with total Akt, total GSK3β, phospho-Akt (Ser473), and phospho-GSK3β (Ser9) antibodies. In the DMII and DMII + CAF samples, wild type Akt and GSK3β were expressed at similar levels. DMII stimulation of 3T3-L1 adipocytes resulted in a significant increase in phospho-Akt and phospho-GSK3β (Figure 3A and 3B). In the presence of CAF, the expression of phospho-Akt was dramatically decreased in 3T3-L1 adipocytes (Figure 3A). Similar to these findings, the expression of phospho-GSK3β during 3T3-L1 differentiation was also dramatically decreased by CAF treatment (Figure 3B). These results demonstrate that CAF treatment inhibits the phosphorylation of Akt, which suppresses the phosphorylation of its substrate kinase GSK3β.

### CAF inhibits lipid accumulation and induces lipolysis in 3T3-L1 adipocytes

**Figure 4:**
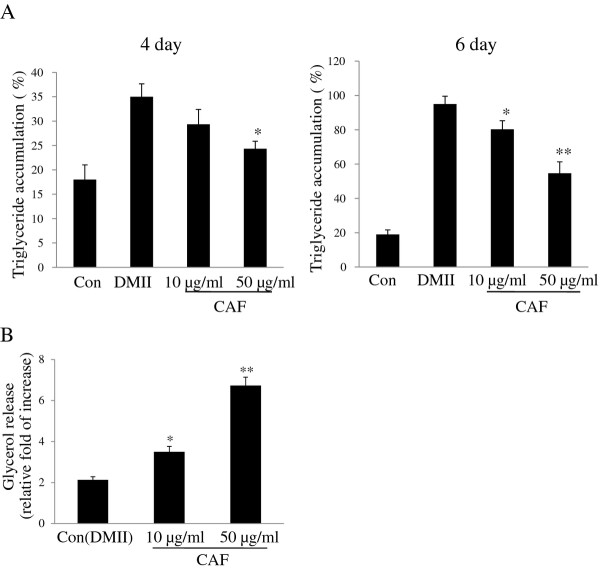
**Effect of CAF on triglyceride accumulation and lypolytic activity in 3T3-L1 adipocytes**. Confluent 3T3-L1 preadipocytes were differentiated into adipocytes in DMII medium for 6 days. (A) CAF reduced TG content during differentiation of 3T3-L1 cells. 3T3-L1 preadipocytes were differentiated in the absence or presence of CAF for 4 or 6 days, and the lipid accumulation was measured by triglyceride assay. Data are mean ± SD values of at least three independent experiments. (*) *p *< 0.05, (**) *P *< 0.01 compared with the differentiated adipocytes (control). (B) CAF increased lipolytic activity in 3T3-L1 adipocytes. The lypolytic activity of CAF was determined by measuring glycerol levels secreted in medium. All experiments were performed on triplicates for each treatment. Values represent mean ± SD. (*) *p *< 0.05, (**) *P *< 0.01 compared with the untreated adipocytes (control).

To explore the effect of CAF on the inhibition of intracellular triglyceride accumulation, 3T3-L1 preadipocytes were differentiated with CAF for 6 days. Lipid accumulation, which was used as a major marker of adipogenesis, was quantified on day 4 and day 6 of the differentiation period. The results show that treatment of CAF reduces triglyceride content and that the inhibition of intracellular triglyceride accumulation in 3T3-L1 adipocytes occurs in a dose-dependent manner (Figure 4A). We then investigated whether the reduction in triglyceride content was associated with lipolysis. 3T3-L1 adipocytes were treated with CAF concentrations of 0, 10, and 50 μg/ml. The lipolytic activity of CAF was determined by measuring the glycerol levels that were released in the medium. Fully differentiated 3T3-L1 adipocytes were treated with DMII every two days over the course of 6 days, and the cells were then treated with medium containing CAF for 24 hours. The small amount of glycerol released into the media was observed in 3T3-L1 adipocytes treated with DMII. However, CAF treatment increased glycerol secretion in a dose-dependent manner, which indicated that CAF strongly stimulated lipolysis in differentiated 3T3-L1 adipocytes (Figure 4B).

## Discussion

In the present study, we examined the anti-obesity effect of CAF in 3T3-L1 cells by measuring lipid accumulation and by analyzing changes in adipocyte differentiation, which modulates adipocyte-specific gene expression and Akt phosphorylation. We demonstrated that CAF treatment inhibited lipid accumulation and the differentiation of 3T3-L1 preadipocytes into adipocytes in a dose-dependent manner. CAF treatment decreased the expression of key adipocyte differentiation regulators, including C/EBPβ, C/EBPα and PPARγ, and down-regulated Akt phosphorylation. These results suggest that CAF plays a critical role in preventing adipogenesis and the accumulation of cytoplasmic lipid droplets during the differentiation in 3T3-L1 cells.

Adipocyte differentiation and fat accumulation are associated with the occurrence and development of obesity [24]. Hyperplastic obesity is caused by an increase in the number of fat cells relative to the increase in adipose tissue mass. A reduction of adiposity is related to the inhibition of angiogenesis along with a reduction of adipocyte numbers and the lipid content of adipocytes. The differentiation of preadipocytes into adipocytes is regulated by a complex network of transcription factors. At the center of this network are nuclear receptor PPARγ and members of C/EBP family, which initiate the entire adipogenic program and regulate the process of terminal differentiation [7]. The expression of C/EPBα and PPARγ cross-regulate each other through a positive feedback loop and transactivate downstream target genes, such as aP2, LPL, and FAS, that are adipocyte specific and are involved in maintaining the adipocyte phenotype [6,9]. In the present study, CAF treatment remarkably reduced the level of Oil-red O staining in a dose-dependent manner, and microscopic inspection revealed a significant decrease in the level of accumulated intracellular triglyceride. The triglyceride levels of cells treated with 50 μg/ml CAF displayed a marked reduction in adipogenesis. These results indicate that CAF inhibited the differentiation of 3T3-L1 preadipocytes into adipocytes and also inhibited the accumulation of lipid droplets in the cytoplasm, an adipocyte phenotype that follows differentiation based on lipid accumulation. Our results show that CAF treatment significantly down-regulated PPARγ and C/EBPβ at the mRNA and protein levels, compared to those in fully differentiated adipocytes. PPARγ and C/EBPβ, which are two central transcriptional regulators, are induced prior to the transcriptional activation of most adipocyte-specific genes [7]. Moreover, PPARγ-deficient cells fail to differentiate into adipocytes, and the overexpression of PPARγ and C/EBPα accelerates adipogenesis [25]. Therefore, these results suggest that CAF suppresses adipocyte differentiation through PPARγ and C/EBPβ in the early stages of adipocyte differentiation.

We focused on the effects of CAF on the regulation of aP2 and FAS expression during 3T3-L1 differentiation. PPARγ and C/EBPα synergistically activate the downstream promoters of adipocyte-specific genes such as aP2 and FAS. The aP2 gene is a terminal differentiation maker of adipocytes, and it facilitates the cellular uptake of long-chain fatty acids in a pathway linking fatty acid metabolism and obesity [26]. FAS is a lipogenic enzyme that facilitates the synthesis and cytoplasmic storage of massive amounts of triglycerides [27]. In the current study, the presence of CAF suppressed the expression of aP2 and FAS, which suggested that CAF inhibits adipogenesis through the down-regulation of C/EBPα and PPARγ. Furthermore, the down-regulation of aP2 and FAS decreased fatty acid utilization and fatty acid transport in 3T3-L1 adipocytes. However, we did not observe any change in the expression of LPL, which is also controlled by PPARγ and C/EBPα. Consistent with our results, hydroxytyrosol from olive oil inhibited lipid accumulation during adipocyte differentiation and inhibited the expression of all genes tested, except LPL [28]. CAF stimulated lipolysis, which induced glycerol release when added to mature adipocytes, correlated to the CAF-induced down-regulation of adipogenic gene expression. Therefore, these results strongly suggest that CAF prevents adipogenesis through the inhibition of PPARγ and C/EBPα gene expression, reduces the expression of adipogenesis- and lipid metabolism-associated genes, and strongly induces lipolysis in 3T3-L1 adipocytes.

Citrus fruits are abundant sources of compounds that help prevent lifestyle-related diseases such as diabetes, high blood pressure, and cancer. CAL contains many flavonoid components, such as naringin, hesperidin, poncirin, isosinnesetin, hexamethoxyflavone, sinestin, nobletin, heptamethoxyflavone, tangeretins, and hydroxypentamethoxyflavone [22,23]. Polymethoxylated flavones from citrus improve lipid and glucose homeostasis and increase adiponectin in fructose-induced insulin-resistant models [29]. *Citrus depressa *Hayata extracts show antiobesity effects in high-fat diet-induced obese mice [30]. Other studies have reported that dietary flavonoids, which include catechin, quercetin, kaempferol, and genistein, inhibit adipogenesis in 3T3-L1 adipocytes [29,31]. Mercader et al. showed that a *Citrus aurantium *extract exhibited lipolytic activity in human adipocytes, providing the basis for an anti-obesity effect [20].

Many studies using natural substances and herbal compounds focus on the activity of the PI3K/Akt signaling pathway in preventing obesity; hormones and growth factors that are specific to adipogenesis act via their receptors to transduce external differentiation signals through a cascade of intracellular events in the PI3K/Akt signaling pathway [32]. Thus, Akt activation has been identified as a major target for the control of obesity and diabetes [12]. Akt plays a critical role in the insulin signaling pathway, and the insulin-stimulated phosphorylation of Akt via PI3K is an important indicator of proper insulin function [13]. Constitutively active Akt causes the spontaneous differentiation of 3T3-L1 cells in the absence of insulin stimulation [12]. The Akt signal cascade is important for adipogenesis, and it activates PPARγ and C/EBPα during 3T3-L1 adipocyte differentiation [11]. Moreover, Akt regulates adipogenesis via the phosphorylation and inactivation of substrates such as Foxo1 and GSK3β, which directly regulate PPARγ, C/EBPβ, C/EBPα, and GS [15,16]. Therefore, to investigate the molecular mechanism underlying the anti-adipogenesis stimulated by CAF, we studied the effects of CAF on the activation of Akt. Our results demonstrate that CAF caused a marked and dose-dependent attenuation of the Akt phosphorylation (Ser473) induced by insulin. However, DMII induction of the 3T3-L1 cells increased Akt activation, which is consistent with enhanced Akt phosphorylation. Our results strongly support the conclusions of a previous study showing that naringenin, which is derived from *Citrus aurantium*, inhibits phosphotidylinositide-3-kinase activity and glucose uptake in 3T3-L1 adipocytes [33]. Intriguing, CAF also decreased insulin-induced GSK3β (Ser9) phosphorylation in a dose-dependent manner in 3T3-L1 adipocytes. The results of another study demonstrated that the Ser9 phosphorylation of GSK3β is increased following insulin treatment, and its activity is repressed by insulin and *lithium chloride *(LC) [33]. Lithium mimics insulin in its stimulation of glucose transport. LC treatment of 3T3-L1 cells inhibited PPARγ expression and adipocyte differentiation [34]. In addition, a study of Akt-deletion mice showed that Akt is essential for adipocyte differentiation and for the induction of PPARγ expression [35]. Therefore, our results indicate that the inhibition of Akt phosphorylation and activation by CAF blocks hormone-induced adipocyte differentiation in 3T3-L1 preadipocytes. These results imply that there is an important association between PI3K/Akt/GSK3β-mediated signaling and the transcription factors, PPARγ and C/EBPα, in 3T3-L1 adipocyte differentiation induction. These results identify one possible mechanism of CAF action, suggesting that CAF-induced inhibition of Akt suppresses adipogenesis by inhibiting other signaling cascades that include C/EBPs and PPARγ during the process of 3T3-L1 adipocyte differentiation.

## Conclusions

In conclusion, we showed that CAF suppressed adipogenesis by down-regulating the expression of PPARγ and C/EBPα as well as by down-regulating the expression of genes that are relevant to lipid accumulation and lipid metabolism. CAF also inhibited adipocyte differentiation in 3T3-L1 adipocytes by attenuating the Akt/GSK3β pathway and by promoting lipolysis of mature adipocytes. Taken together, our findings provide important insights into the mechanisms underlying the anti-obesity activity of CAF.

## Abbreviations

CAL: Citrus aurantium L.; CAF: Citrus aurantium flavonoid; PPARγ: Peroxisome proliferating-activated receptor-gamma; C/EBP-α: CCAAT-enhancer binding protein-alpha; C/EBP-β: CCAAT-enhancer binding protein-beta; LPL: Lipoprotein lipase; FAS: Fatty acid synthase

## Competing interests

The authors declare that they have no competing interests.

## Authors' contributions

GSK, HJP, JHW, and MKK performed a chemical assay and cell biology studies of cultured cells. GSK, HJP, POK, WM, YGK, CHK, CKW and JHC conceived the idea, designed the experiments, and interpreted the experimental results. All authors contributed to manuscript preparations and approved the final manuscript.

## Pre-publication history

The pre-publication history for this paper can be accessed here:

http://www.biomedcentral.com/1472-6882/12/31/prepub
